# In Vitro Effects of *Weissella cibaria* CMU and CMS1 on Receptor Activator of NF-κB Ligand (RANKL)-Induced Osteoclast Differentiation

**DOI:** 10.3390/jfb15030065

**Published:** 2024-03-08

**Authors:** Geun-Yeong Park, Jeong-Ae Park, Mi-Sun Kang

**Affiliations:** R&D Center, OraTicx, Inc., Seoul 04782, Republic of Korea; gypark@oraticx.com (G.-Y.P.); pja@oraticx.com (J.-A.P.)

**Keywords:** *Weissella cibaria*, RAW 264.7 cells, osteoclast, bone resorption, periodontitis

## Abstract

Excessive osteoclast activity can promote periodontitis-associated bone destruction. The inhibitory mechanisms of *Weissella cibaria* strains CMU and CMS1 against periodontitis have not yet been fully elucidated. In this study, we aimed to investigate whether heat-killed (HK) *W. cibaria* CMU and CMS1 or their respective cell-free supernatants (CFSs) inhibit osteoclast differentiation and bone resorption in response to receptor activator of nuclear factor kappa-B ligand (RANKL)-treated RAW 264.7 cells. TRAP (tartrate-resistant acid phosphatase) staining and bone resorption assays revealed that both HK bacteria and CFSs significantly suppressed the number of TRAP-positive cells, TRAP activity, and bone pit formation compared to the RANKL-treated control (*p* < 0.05). HK bacteria dose-dependently inhibited osteoclastogenesis while selectively regulating certain genes in CFSs (*p* < 0.05). We found that disrupting the direct interaction between HK bacteria and RAW 264.7 cells abolished the inhibitory effect of HK bacteria on the expression of osteoclastogenesis-associated proteins (c-Fos, nuclear factor of activated T cells c1 (NFATc1), and cathepsin K). These results suggest that dead bacteria suppress osteoclast differentiation more effectively than the metabolites and may serve as beneficial agents in preventing periodontitis by inhibiting osteoclast differentiation via direct interaction with cells.

## 1. Introduction

Periodontal disease is a prevalent and widespread chronic inflammatory disease that affects approximately 20–50% of the global population and encompasses gingivitis and periodontitis [1,2]. Moreover, periodontitis is a major cause of tooth loss. Upon infection with periodontal bacteria, pro-inflammatory cytokines increase, and inflammation occurs around the gums. The inflammatory response activates osteoclasts and destroys the alveolar bone near the gums, thereby compromising the capacity of the bone to support teeth, leading to tooth loss [3].

Osteoclasts regulate bone metabolism together with osteoblasts, another type of bone cell [4,5,6]. Osteoclasts and osteoblasts participate in bone matrix formation, and when periodontitis occurs, osteoclasts can be activated to resorb and decompose the bone. Receptor activator of nuclear factor (NF)-κB ligand (RANKL) is a cell signaling protein found in osteoblasts [7]. RANKL binds to RANK present in osteoclast precursors, activating the mitogen-activated protein kinase (MAPK) and NF-κB signaling pathways and sequentially activating nuclear factor of activated T cells c1 (NFATc1), which is essential for osteoclast differentiation [8,9]. Furthermore, it is known to promote osteoclast differentiation and bone resorption by inducing the expression of several genes [9,10,11,12,13]. Therefore, inflammatory periodontal disease associated with osteoclasts can be alleviated by suppressing osteoclast differentiation [14].

Effective treatments for managing periodontal disease are limited, and researchers are continually developing treatments to prevent periodontitis alongside oral hygiene management. Recently, as the importance of disease prevention has been emphasized worldwide, probiotics have been proposed as an alternative for periodontal disease prevention [15,16,17,18]. Given the crucial role of the oral microbiota in regulating alveolar bone remodeling [15], oral probiotics have been explored as a potential therapeutic intervention to maintain alveolar bone homeostasis and prevent periodontitis. Representative studies have been conducted employing various probiotics, including *Bifidobacterium*, *Lactobacillus*, and *Weissella* [17,18,19,20,21,22,23,24,25,26,27,28].

*Weissella cibaria* strains CMU and CMS1 were isolated from the saliva of children with good oral health and Gram-positive lactic acid bacteria with a short rod-shaped morphology [19]. Both animal and clinical studies, including in vitro evaluations, indicate that these strains can inhibit the progression of periodontitis by regulating the inflammatory response of host cells [18,19,20,21,22]. Kim et al. [18] demonstrated that bone loss in periodontitis-induced mice was prevented by treatment with the oral probiotic *W. cibaria* CMU, highlighting its capacity to reduce inflammatory cytokines in gum tissue. In addition, it has been proven that *W. cibaria* CMU inhibits the formation of pro-inflammatory cytokines in human gingival fibroblasts caused by periodontal disease-causing bacteria such as *Fusobacterium nucleatum*, *Prevotella intermedia*, and *Porphyromonas gingivalis* [22]. However, the effects of *W. cibaria* CMU and CMS1 on RANKL-induced osteoclast differentiation remain unexplored.

The murine macrophage cell line RAW 264.7 stands out as the primary host cell line for studying osteoclast differentiation owing to its pronounced expression of RANK in response to RANKL [29,30]. Several studies have reported that various probiotic bacterial strains inhibit RANK-induced osteoclastogenesis in RAW 264.7 cells [23,24,25]. Therefore, in this study, we evaluated the effects of oral probiotics *W. cibaria* CMU and CMS1 on RANKL-stimulated osteoclast differentiation in RAW 264.7 cells in vitro by elucidating the molecular mechanisms underlying osteogenesis inhibition.

## 2. Materials and Methods

### 2.1. Cell Culture and Osteoclast Differentiation In Vitro

The RAW 264.7 cell line (murine macrophage) was purchased from the Korean Cell Line Bank (KCLB, Seoul, Republic of Korea). Cells were grown in Dulbecco’s Modified Eagle Medium (DMEM; Gibco, Thermo Fisher Scientific, Gaithersburg, MD, USA) supplemented with 10% heat-inactivated fetal bovine serum (FBS; Gibco) and 1% antibiotic–antimycotic solution (GenDepot, Katy, TX, USA) at 37 °C in a 5% CO_2_ humidified atmosphere. Experiments were conducted on cell passages 2 to 10. The cells were subcultured and plated at 80% confluency. For osteoclast differentiation, alpha-minimal essential medium (α-MEM; Welgene Inc., Daegu, Republic of Korea) containing 10% FBS was treated with RANKL (100 ng/mL; Peprotech, Cranbury, NJ, USA). The medium was changed every alternate day throughout the culture period.

### 2.2. Preparation of Heat-Killed Bacteria and Cell-Free Supernatants

*W. cibaria* CMU (oraCMU) and CMS1 (oraCMS1) were grown aerobically in DeMan, Rogosa, and Sharpe (MRS) broth (Difco, Detroit, MI, USA) at 37 °C for 16 h. To prepare the heat-killed (HK) bacteria, the cultures were centrifuged (5000× *g*, 10 min, 4 °C), the resulting pellets were washed twice with phosphate-buffered saline (PBS), resuspended in DMEM, and the concentration was adjusted to an optical density (OD) of 0.5 at 600 nm (approximately 5 × 10^8^ CFU/mL). The bacteria were then exposed to heat (110 °C) for 10 min. Cell-free supernatants (CFSs) were prepared by centrifuging the culture for 24 h, followed by filtration (0.22 μm; JET BIOFIL, Guangzhou, China) to remove cells. The CFSs were lyophilized, resuspended in DMEM, and filtered again (0.45 μm; JET BIOFIL).

### 2.3. Cell Viability Assay

A viability assay kit (Cellrix, MediFab, Seoul, Republic of Korea) was used to measure cell viability after treatment with test substances (HK-oraCMU and HK-oraCMS1 or CFS-oraCMU and CFS-oraCMS1). RAW 264.7 cells were seeded on 96-well plates (1 × 10^4^ cells/well) and incubated at 37 °C for 16 h. The culture medium used to induce the differentiation of RAW 264.7 cells into osteoclasts were replaced with α-MEM supplemented with 10% FBS and incubated with RANKL at various concentrations of HK bacteria (multiplicity of infection (MOI) = 1, 10, 100, or 1000) or CFSs (0.25, 0.5, 1, or 2 mg/mL) for 2 d. Subsequently, the culture was removed and carefully replaced with fresh medium containing a water-soluble tetrazolium-8 (WST-8) salt solution and incubated for 4 h at 37 °C under 5% CO_2_. Cell viability was measured at 450 nm using a microplate reader (VersaMax, Molecular Devices, San Jose, CA, USA) and expressed as a percentage relative to the untreated negative control.

### 2.4. Tartrate-Resistant Acid Phosphatase (TRAP) Staining and Activity

RAW 264.7 cells were seeded on a 96-well plate (1 × 10^4^ cells/well) for 16 h and incubated for an additional 5 days in an induction medium containing RANKL and various concentrations of the test substances. The induction medium was replaced every other day. TRAP staining and activity assays were performed according to the manufacturer’s instructions (Cosmo Bio, Tokyo, Japan) to detect osteoclasts. Briefly, the culture medium was removed, and the cells were washed with PBS, and fixed with 10% formalin neutral buffer for 5 min at 25 °C. Each well was washed three times with distilled water (DW), and chromogenic substrate in tartrate-containing buffer was added to each well and incubated for 60 min at 37 °C. The wells were washed again three times with DW and dried at 25 °C for 2 h. Stained cells were observed under an inverted microscope (OLYMPUS CKX53, Olympus, Tokyo, Japan), and multinucleated cells containing three or more nuclei were identified as osteoclasts. To evaluate TRAP activity, 30 μL of the collected culture medium described above was transferred to a new well, mixed with 170 μL of chromogenic substrate in tartrate-containing buffer (Cosmo Bio), and incubated at 37 °C for 3 h. TRAP activity was assessed at 540 nm, and the results were expressed as a percentage of the control (RANKL-treated only).

### 2.5. Bone Resorption Assay

The effect of the test substances on osteoclast-mediated bone resorption was determined using dentin discs (Immunodiagnostic Systems, Boldon, UK). RAW 264.7 cells were seeded on a 96-well plate (5 × 10^3^ cells/well) for 16 h and incubated for an additional 5 days in the induction medium containing RANKL and various concentrations of the test substances. The induction medium was replaced every alternate day. The medium was removed, treated with 5% sodium hypochlorite for 5 min, and washed three times with DW. Toluidine blue solution (0.1%; Sigma-Aldrich, St. Louis, MO, USA) was added to each well for 3 min, washed again with DW three times, dried at 25 °C for 2 h, and observed under an inverted microscope at 400× magnification. Image J software Ver. 1.54 (National Institutes of Health, Washington, DC, USA) was used to measure the total pit area. The pit area was expressed as a value relative to that of the control (RANKL-treated only).

### 2.6. Reverse Transcription (RT)-Quantitative Polymerase Chain Reaction (qPCR)

RT-qPCR was performed to investigate the effect of the test substances on the expression of osteoclast differentiation-mediated genes. RAW 264.7 cells were seeded on a 6-well plate (1 × 10^5^ cells/well), and the following day, the cells were replaced with induction medium containing RANKL and various concentrations of the test substances and subsequently cultured for an additional 2 days. Total RNA was extracted, and RT-qPCR was performed on a Rotor Gene Q system (Qiagen, Hilden, Germany) using the PrimeScript RT kit (Takara Bio, Shiga, Japan) and the PowerUp SYBR Green PCR Master Mix (Applied Biosystems, Thermo Fisher Scientific) as previously described [22]. The primer sequences were as follows: mouse *cathepsin K* forward (F), 5′-GAAGAAGACTCACCAGAAGCAG-3′ and reverse (R), 5′-TCCAGG TTATGGGCAGAGATT-3′; mouse *c-Fos* F, 5′-CGGGTTTCAACGCCGACTA-3′ and R, 5′-TGGCACTAGAGACGGACAGAT-3′; mouse *NFATc1* F, 5′-GGTGCTGTCTGG CCATAACT-3′ and R, 5′-GCGGAAAGGTGGTATCTCAA-3′; mouse *TRAP* F, 5′-GACAAGAGGTTCCAGGAGACC-3′; and R, 5′-GGGCTGGGGAAGTTCCAG-3′; mouse *osteoclast associated Ig-like receptor* (*OSCAR*) F, 5′-CTGCTGGTAACGGATCAGCT CCCCAGA-3′ and R, 5′-CCAAGG AGCCAGAACCTTCGAAACT-3′; mouse *dendritic cell specific transmembrane protein* (*DC-STAMP*) F, 5′-CCAAGGAGTCGTCCATGATT-3′ and R, 5′-GGCTGCTTTGATCGTTTCTC-3′; mouse *glyceraldehyde 3-phosphate dehydrogenase* (*GAPDH*) F, 5′-AGGTCGGTGTGAACGGATTTG-3′ and R, 5′-TGTAGACCATGTAGTTGAGGTCA-3′. Relative mRNA expression values were obtained by the 2^−ΔΔCT^ method, and relative gene expression was normalized to GAPDH expression.

### 2.7. Western Blotting Analyses

RAW 264.7 cells were seeded on a 6-well plate (1 × 10^5^ cells/well), and the following day, the cells were replaced with induction medium containing RANKL and various concentrations of the test substances and subsequently cultured for an additional 2 days. After cell seeding, a cell culture insert (pore size: 0.4 µm; SPL Life Sciences, Pocheon, Republic of Korea) was placed onto the relevant well, and HK *W. cibaria* was added onto the filter membrane. After incubation, cells were washed once with ice-cold Dulbecco’s PBS (GenDepot), and proteins were extracted using an EzRIPA Lysis kit (ATTO, Tokyo, Japan), according to the manufacturer’s protocol. Proteins (25 μg) were resolved by SDS–PAGE on 10% acrylamide gels and transferred onto polyvinylidene fluoride membranes (0.45 μm; Amersham, Cytiva, Marlborough, MA, USA). Membranes were blocked with 5% skim milk in Tris-buffered saline with 0.1% Tween 20 (GenDepot) for 1 h at 25 °C and incubated overnight at 4 °C with the following primary antibodies: c-Fos, NFATc1, cathepsin K, phospho-p44/42 MAPK (p-ERK), ERK, p-IκBα, IκBα, p-p38, p38, p-SAPK/JNK (p-JNK), JNK, and β-actin. Membranes were washed and incubated with horseradish peroxidase-conjugated secondary antibodies at 25 °C for 1 h. c-Fos and NFATc1 were purchased from Santa Cruz Biotechnology (Dallas, TX, USA) and BD Biosciences (San Jose, CA, USA), respectively. All other antibodies were purchased from Cell Signaling Technology (Danvers, MA, USA).

Each protein band was visualized using a West-Q Chemiluminescent Substrate Kit Plus (GenDepot) and imaged using a Chemiluminescence Imaging System (WSE-6200 LuminoGraph II, ATTO). Protein expression was quantified using CSAnalyzer4 version 2.4.5 (ATTO), and the relative expression was normalized to β-actin expression.

### 2.8. Statistical Analysis

The data were expressed as the mean ± SD of three independent experiments. SPSS Statistics version 21.0 for Windows (IBM, Armonk, NY, USA) was used for statistical analysis. To assess differences between group means, one-way analysis of variance (ANOVA) or Welch’s ANOVA calculations with the Duncan multiple test or Games–Howell post-hoc test were performed. The statistical significance level of *p* < 0.05 was adopted.

## 3. Results

### 3.1. Cytotoxicity of W. cibaria CMU and CMS1 in RAW 264.7 Cells

**Figure 1:**
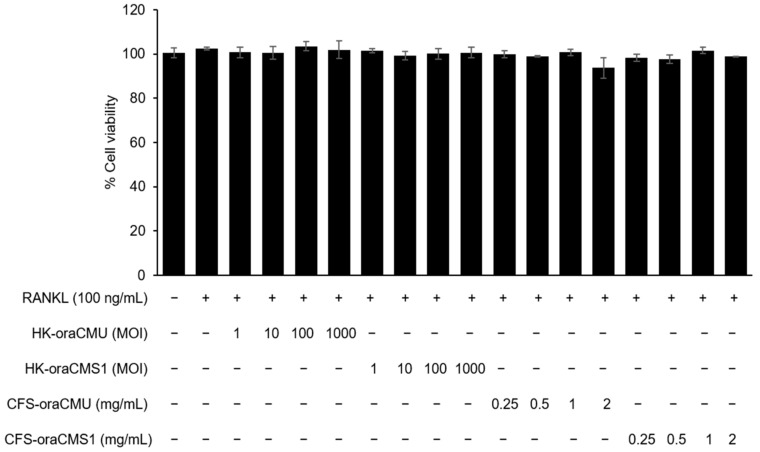
Effects of heat-killed *W. cibaria* CMU (HK-oraCMU), CMS1 (HK-oraCMS1), or the cell-free supernatants (CFSs) of *W. cibaria* CMU (CFS-oraCMU) and CMS1 (CFS-oraCMS1) on the viability of RAW 264.7 cells. All groups showed no statistically significant differences between groups (*p* > 0.05).

During the differentiation process into RANKL-treated osteoclasts, high concentrations of HK *W. cibaria* CMU (HK-oraCMU) and CMS1 (HK-oraCMS1) (MOI = 1000) did not reduce cell viability, and the CFSs of *W. cibaria* CMU (CFS-oraCMU) and CMS1 (CFS-oraCMS1) did not reduce cell viability even at high concentrations (2 mg/mL) (Figure 1).

### 3.2. Effects of W. cibaria CMU and CMS1 on RANKL-Induced Osteoclastogenesis

#### 3.2.1. HK *W. cibaria* Inhibited Osteoclastogenesis and TRAP Activity in RAW 264.7 Cells

**Figure 2:**
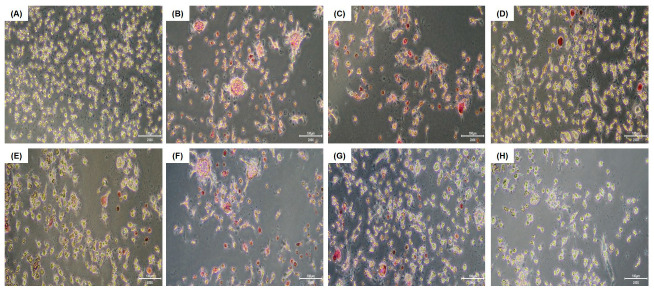

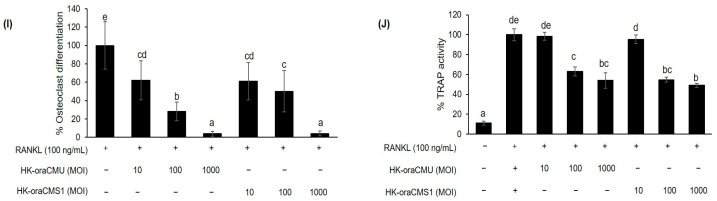
Dose-dependent inhibition of osteoclast differentiation by HK-oraCMU and HK-oraCMS1. RAW 264.7 cells were cultured in the presence of RANKL (100 ng/mL) and various concentrations of HK *W. cibaria* for 5 days. TRAP staining was observed using a microscope (scale bars = 100 μm; magnification: ×200). (**A**–**H**) Representative images of TRAP staining. (**A**) Untreated negative control; (**B**) RANKL-treated control; (**C**–**E**) RANKL + HK-oraCMU (MOI = 10, 100, or 1000); (**F**–**H**) RANKL + HK-oraCMS1 (MOI = 10, 100, or 1000); (**I**) giant multinucleated cells containing ≥ 3 nuclei that stained positive for TRAP were identified as osteoclasts. Osteoclast differentiation was expressed as a percentage of the control (RANKL-treated only); (**J**) TRAP activity was determined at 540 nm. TRAP activity was expressed as a percentage of the control (RANKL-treated only). HK-oraCMU, heat-killed *W. cibaria* CMU; HK-oraCMS1, heat-killed *W. cibaria* CMS1. Different alphabet letters (a–e) indicate statistical differences as determined by ANOVA (*p* < 0.05).

TRAP-positive, stained multinucleated cells were identified as osteoclasts (Figure 2). It was observed that RANKL induced RAW 264.7 cells to differentiate into mature multinucleated osteoclasts; however, the treatment with HK-oraCMU and HK-oraCMS1 significantly reduced osteoclast formation in a dose-dependent manner (*p* < 0.05) (Figure 2I). High concentrations of HK-oraCMU and HK-oraCMS1 (MOI = 1000) reduced osteoclast differentiation by 96.1% and 95.7%, respectively. TRAP activity was significantly increased by RANKL, and conversely, it was significantly decreased in the HK-oraCMU and HK-oraCMS1 treatment groups dose-dependently (*p* < 0.05) (Figure 2J). High concentrations of HK-oraCMU and HK-oraCMS1 (MOI = 1000) reduced TRAP activity by 46.0% and 50.9%, respectively.

#### 3.2.2. CFSs of *W. cibaria* Inhibited Osteoclastogenesis and TRAP Activity in RAW 264.7 Cells

**Figure 3:**
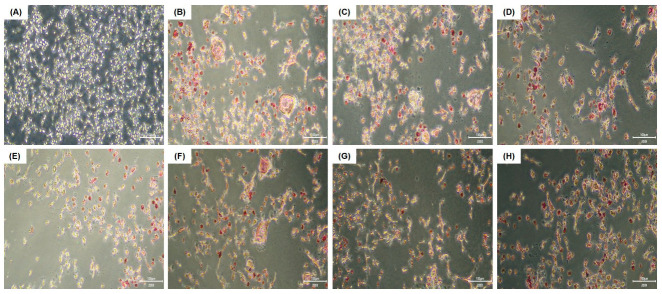

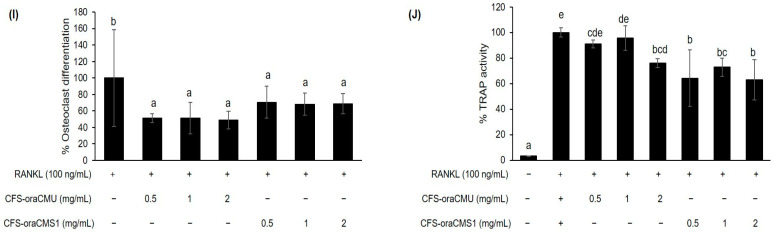
Inhibitory effects of the CFS-oraCMU and CFS-oraCMS1 on osteoclast differentiation. RAW 264.7 cells were incubated with RANKL (100 ng/mL) and various concentrations of CFS-oraCMU or CFS-oraCMS1 for 5 days. TRAP staining was observed using a microscope (scale bars = 100 μm; magnification: ×200). (**A**–**H**) Representative images of TRAP staining. (**A**) Untreated negative control; (**B**) RANKL-treated control; (**C**–**E**) RANKL + CFS-oraCMU (0.5, 1, or 2 mg/mL); (**F**–**H**) RANKL + CFS-oraCMS1 (0.5, 1, or 2 mg/mL); (**I**) giant multinucleated cells containing ≥ 3 nuclei that stained positive for TRAP were identified as osteoclasts. Osteoclast differentiation was expressed as a percentage of the control (RANKL-treated only); (**J**) TRAP activity was determined at 540 nm. TRAP activity was expressed as a percentage of the control (RANKL-treated only). CFS-oraCMU, cell-free supernatants of *W. cibaria* CMU; CFS-oraCSM1, cell-free supernatants of *W. cibaria* CMS1. Different alphabet letters (a–e) indicate statistical differences as determined by ANOVA (*p* < 0.05).

The effects of the CFS-oraCMU and CFS-oraCMS1 treatments on osteoclast differentiation are presented in Figure 3. Compared to the RANKL alone group, treatment with both CFS-oraCMU and CFS-oraCMS1 significantly inhibited differentiation into TRAP-positive multinucleated osteoclasts (*p* < 0.05) (Figure 3I). High concentrations of CFS-oraCMU and CFS-oraCMS1 (2 mg/mL) reduced osteoclast differentiation by 51.2% and 31.9%, respectively. Moreover, TRAP activity significantly decreased only in the high-concentration CFS-oraCMU treatment group (2 mg/mL), while all CFS-oraCMS1-treated groups exhibited decreased TRAP activity (Figure 3J). High concentrations of CFS-oraCMU and CFS-oraCMS1 reduced TRAP activity by 24.0% and 36.9%, respectively.

### 3.3. Effects of W. cibaria CMU and CMS1 on Bone Resorption

**Figure 4:**
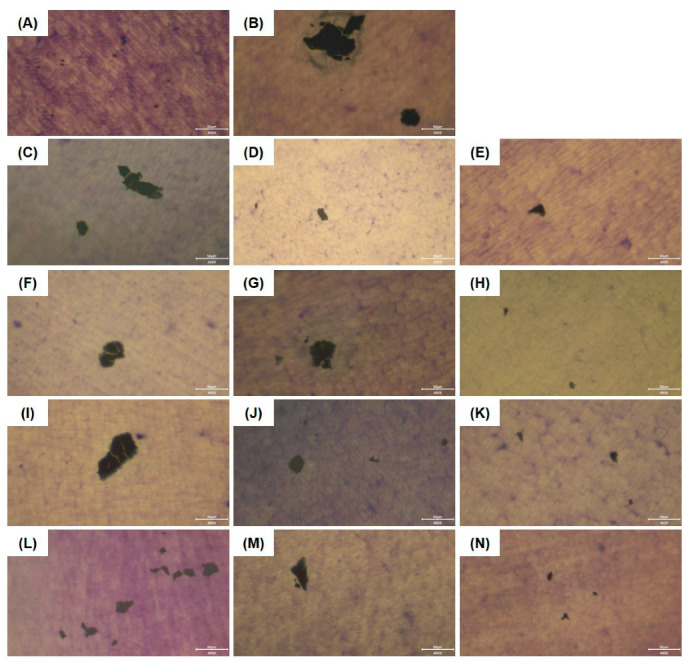

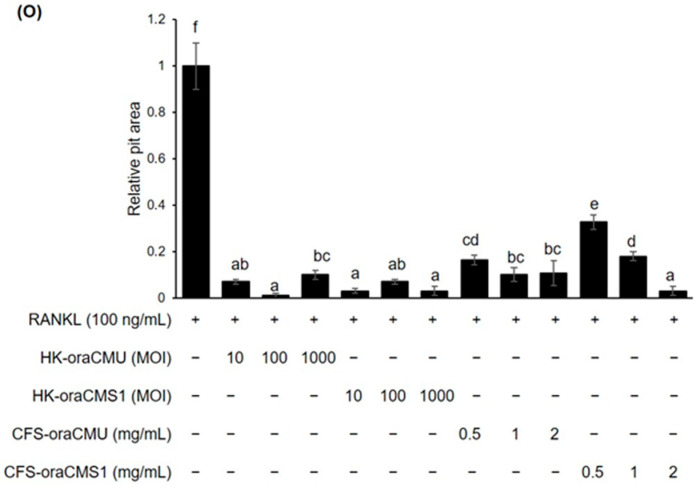
Inhibitory effects of *W. cibaria* CMU and CMS1 on bone resorption. (**A**–**N**) Representative images of the bone resorption pit. RAW 264.7 cells were incubated with RANKL (100 ng/mL) and various concentrations of the test substances for 5 days. Bone resorption was observed using a microscope (scale bars = 50 μm; magnification: ×400). When the bone was resorbed, a pit was formed, which appeared black. (**A**) Untreated negative control; (**B**) RANKL-treated control; (**C**–**E**) RANKL + HK-oraCMU (MOI = 10, 100, or 1000); (**F**–**H**) RANKL + HK-oraCMS1 (MOI = 10, 100, or 1000); (**I**–**K**) RANKL + CFS-oraCMU (0.5, 1, or 2 mg/mL); (**L**–**N**) RANKL + CFS-oraCMS1 (0.5, 1, or 2 mg/mL); (**O**) quantitative analysis of bone resorption pit area. The pit area was expressed as a value relative to the RANKL-treated control. HK-oraCMU, heat-killed *W. cibaria* CMU; HK-oraCMS1, heat-killed *W. cibaria* CMS1; CFS-oraCMU, cell-free supernatants of *W. cibaria* CMU; CFS-oraCSM1, cell-free supernatants of *W. cibaria* CMS1. Different alphabet letters (a–f) indicate statistical differences as determined by ANOVA (*p* < 0.05).

To determine whether bone resorption was inhibited by HK *W. cibaria*, pit formation was evaluated microscopically on dentin discs. The dentin section surface of the untreated negative control group appeared clean, whereas the group treated with RANKL alone exhibited large bone resorption pits (Figure 4B). Both HK-oraCMU and HK-oraCMS1 significantly inhibited pit formation by 99.0%, regardless of the concentration used (Figure 4O). Additionally, as shown in Figure 4I–N, pit formation was inhibited in both CFS-oraCMU and oraCMS1 groups compared to the RANKL-only group, and quantitative analysis confirmed that the pit formation was significantly suppressed (67.3–97.0%) in these groups compared to the RANKL-only control group (*p* < 0.05) (Figure 4O).

### 3.4. Effects of W. cibaria CMU and CMS1 on Osteoclastogenesis-Associated Gene Expression

#### 3.4.1. HK *W. cibaria* Suppressed Osteoclastogenesis-Associated Gene Expression

**Figure 5:**
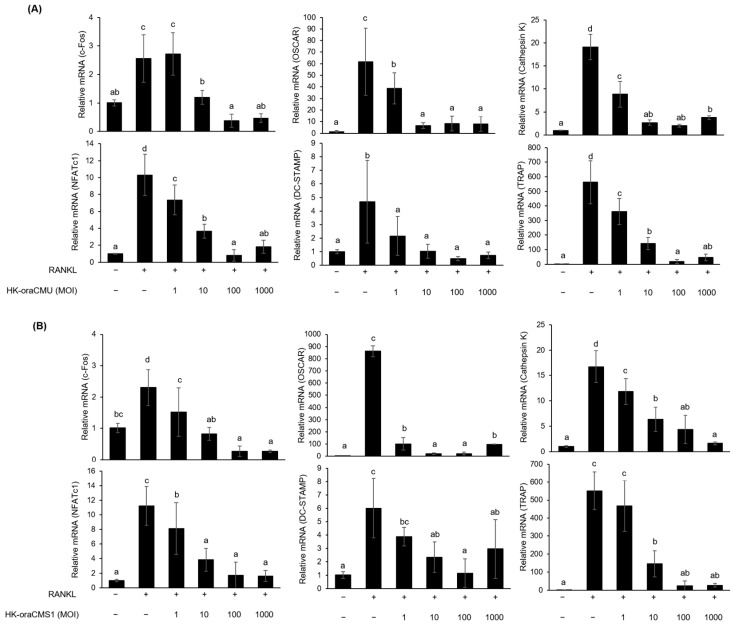
Inhibitory effects of HK-oraCMU (**A**) and HK-oraCMS1 (**B**) on the expression levels of osteoclast differentiation-associated genes in RANKL-stimulated RAW 264.7 cells. RAW 264.7 cells were incubated with RANKL (100 ng/mL) and various concentrations of the test substances for 2 days. HK-oraCMU, heat-killed *W. cibaria* CMU; HK-oraCMS1, heat-killed *W. cibaria* CMS1. Relative gene expression was normalized to that of GAPDH. Different alphabet letters (a–d) indicate statistical differences as determined by ANOVA (*p* < 0.05).

We measured the mRNA expression of osteoclastogenesis-associated genes in RANKL-induced RAW 264.7 cells that were treated with various concentrations of HK *W. cibaria.* RANKL-induced in RAW 264.7 cells increased the mRNA expression of osteoclastogenesis-associated genes compared to that in the untreated negative control (*p* < 0.05) (Figure 5). Both HK-oraCMU and HK-oraCMS1 significantly inhibited the mRNA expression of c-Fos, NFATc1, OSCAR, DC-STAMP, cathepsin K, and TRAP in a dose-dependent manner compared to the RANKL-treated control (Figure 5A,B). In the HK-oraCMU group, the mRNA expression of c-Fos, NFATc1, OSCAR, DC-STAMP, cathepsin K, and TRAP was suppressed by 85.3%, 92.0%, 89.2%, 89.4%, 89.6%, and 96.8%, respectively. Additionally, HK-oraCMS1 suppressed the mRNA expression of c-Fos, NFATc1, OSCAR, DC-STAMP, cathepsin K, and TRAP by 88.7%, 85.3%, 97.5%, 80.9%, 90.3%, and 95.5%, respectively.

#### 3.4.2. CFSs of *W. cibaria* Selectively Suppressed the Expression of Osteoclastogenesis-Associated Genes

**Figure 6:**
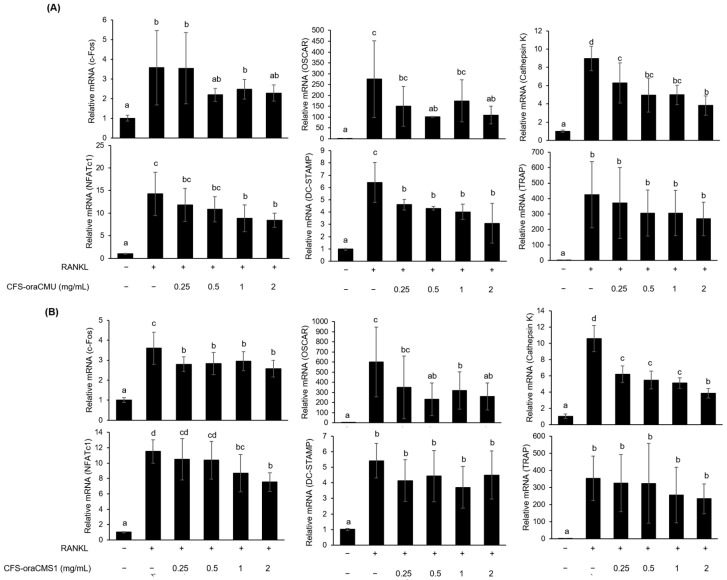
Inhibitory effects of CFS-oraCMU (**A**) and CFS-oraCMS1 (**B**) on the expression levels of osteoclast differentiation-associated genes in RNAKL-induced RAW 264.7 cells. RAW 264.7 cells were incubated with RANKL (100 ng/mL) and various concentrations of the test substances for 2 days. CFS-oraCMU, cell-free supernatants of *W. cibaria* CMU; CFS-oraCMS1, cell-free supernatants of *W. cibaria* CMS1. Relative gene expression was normalized to that of GAPDH. Different alphabet letters (a–d) indicate statistical differences as determined by ANOVA (*p* < 0.05).

The effects of CFS-oraCMU and CFS-oraCMS1 on the mRNA expression of osteoclastogenesis-associated genes are shown in Figure 6. CFS-oraCMU significantly suppressed the mRNA expression of NFATc1, OSCAR, DC-STAMP, and cathepsin K (Figure 6A). CFS-oraCMU inhibited the mRNA expression of NFATc1, OSCAR, DC-STAMP, and cathepsin K by 40.8%, 34.6%, 49.4%, and 57.5%, respectively. Similarly, CFS-oraCMS1 significantly downregulated the mRNA expression of c-Fos, NFATc1, OSCAR, and cathepsin K (Figure 6B). CFS-oraCMS1 suppressed the mRNA expression of c-Fos, NFATc1, OSCAR, and cathepsin K by 30.4%, 38.3%, 61.3%, and 63.5%, respectively.

### 3.5. Effects of W. cibaria CMU and CMS1 on Osteoclastogenesis-Associated Protein Expression

#### 3.5.1. HK *W. cibaria* Suppressed Osteoclastogenesis-Associated Protein Expression

**Figure 7:**
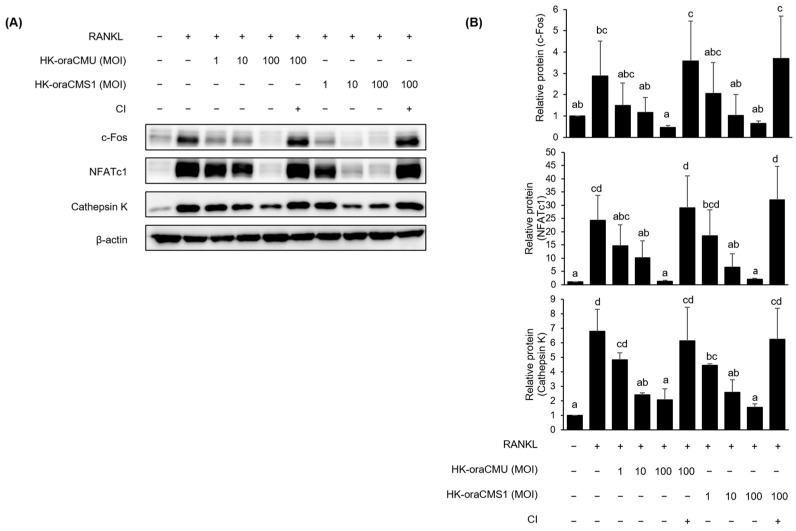
Inhibitory effects of HK-oraCMU and HK-oraCMS1 on the expression of osteoclast differentiation-associated proteins in RANKL-induced RAW 264.7 cells. RAW 264.7 cells were incubated for 2 days with RANKL (100 ng/mL) and various concentrations of the test substances. The protein expression levels were measured by Western blotting (**A**) and quantified (**B**). The quantification of the relative protein expression was normalized to the expression of β-actin. CI—cell culture inserts in the Transwell system. Different alphabet letters (a–d) indicate statistical differences as determined by ANOVA (*p* < 0.05).

We subsequently examined the capacity of HK-oraCMU or HK-oraCMS1 to suppress the expression of osteoclastogenesis-associated proteins through Western blot analysis, with the results presented in Figure 7. RANKL (100 ng/mL) stimulation increased c-Fos, NFATc1, and cathepsin K protein expression in RAW264.7 cells, which was dose-dependently inhibited by treatment with HK-oraCMU or HK-oraCMS1. In contrast, protein expression was not decreased when RAW264.7 cells were cultured without direct contact with HK bacteria using a cell culture insert. When a high concentration of HK-oraCMU or HK-oraCMS1 (MOI = 100) was added onto the filter membrane of the cell culture insert, the expression levels of c-Fos, NFATc1, and cathepsin K were not significantly different from those of the positive control group treated with RANKL alone (Figure 7B). HK-oraCMU inhibited c-Fos, NFATc1, and Cathepsin K protein expression by 84.1%, 94.5%, and 69.5%, respectively, and HK-oraCMS1 inhibited the expression of these proteins by 77.5%, 91.9%, and 76.9%, respectively.

#### 3.5.2. CFSs of *W. cibaria* Suppressed Osteoclastogenesis-Associated Protein Expression

**Figure 8:**
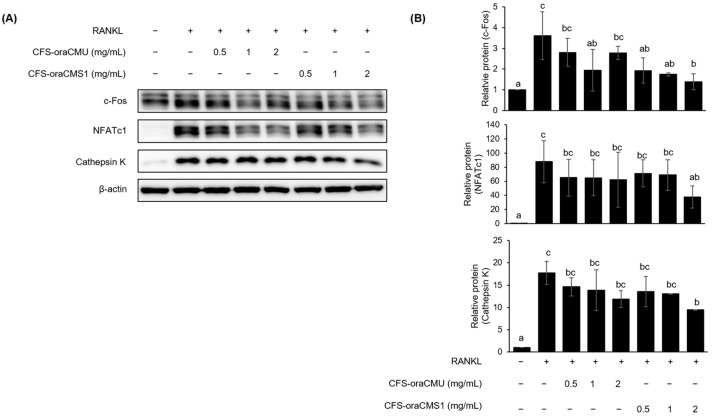
Inhibitory effects of CFS-oraCMU and CFS-oraCMS1 on the expression levels of osteoclast differentiation-associated proteins in RANKL-stimulated RAW 264.7 cells. RAW 264.7 cells were incubated with RANKL (100 ng/mL) and various concentrations of the test substances for 2 days. The protein expression levels were measured by Western blotting (**A**) and quantified (**B**). The protein expression was quantified and normalized to the expression of β-actin. Different alphabet letters (a–c) indicate statistical differences as determined by ANOVA (*p* < 0.05).

We further investigated the effect of CFS-oraCMU or CFS-oraCMU on the expression of osteoclastogenesis-associated proteins in RAW264.7 cells. Treatment with various concentrations of CFS decreased c-Fos, NFATc1, and cathepsin K protein expression (Figure 8). Moreover, it was confirmed that the protein expression level in the group treated with the highest concentration of CFS-oraCMS1 (2 mg/mL) was significantly lower than that in the positive control group treated with RANKL alone (Figure 8B). CFS-oraCMU inhibited c-Fos, NFATc1, and cathepsin K protein expression by 49.8%, 47.6%, and 33.1%, respectively, and CFS-oraCMS1 inhibited the expression of these proteins by 61.3%, 57.2%, and 46.8%, respectively.

### 3.6. Effects of W. cibaria CMU and CMS1 on Cell Signaling Pathways

**Figure 9:**
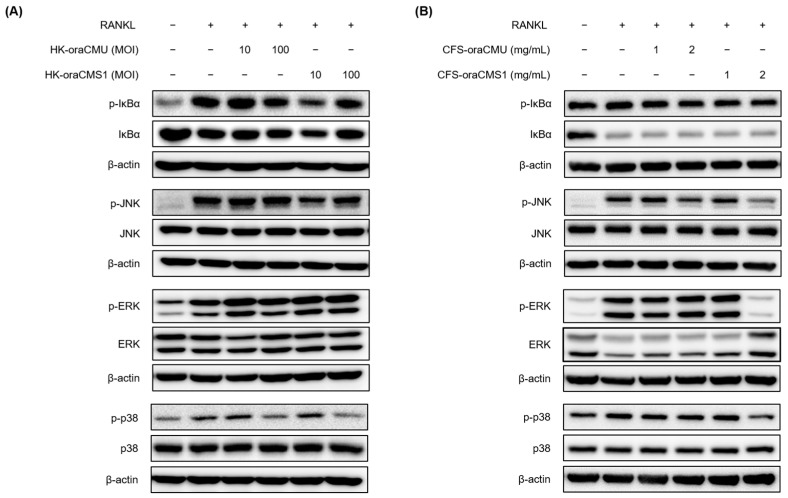
Inhibitory effects of heat-killed (**A**) or cell-free supernatants (**B**) of *W. cibaria* strains CMU and CMS1 on the expression levels of the NF-κB and MAPK signaling pathways in RANKL-stimulated RAW 264.7 cells. RAW 264.7 cells were incubated with RANKL (100 ng/mL) and various concentrations of the test substances. Western blot analysis was used to measure the levels of proteins associated with NF-κB and MAPK activation, including IκBα, p38, JNK, and ERK, at 5 or 15 min. HK-oraCMU, heat-killed *W. cibaria* CMU; HK-oraCMS1, heat-killed *W. cibaria* CMS1; CFS-oraCMU, cell-free supernatants of *W. cibaria* CMU; CFS-oraCMS1, cell-free supernatants of *W. cibaria* CMS1. The relative protein expression was quantified and normalized to the expression of β-actin.

Western blot analysis was performed to investigate whether HK *W. cibaria* or CFSs inhibits the activation of the NF-κB and MAPK pathways. RANKL (100 ng/mL) stimulation increased the phosphorylation of NF-κB and MAPK. Treatment with HK-oraCMU and HK-oraCMS1 for 5 min attenuated IκBα phosphorylation in both strains, and treatment for 15 min dose-dependently attenuated the phosphorylation of JNK and p38, but not ERK (Figure 9A). Following treatment with CFS-oraCMU and CFS-oraCMS1 for 5 min, IκBα phosphorylation was slightly weakened by both strains, and following 15 min of treatment, the phosphorylation of JNK and p38 was reduced in a dose-dependent manner. In addition, treatment with 2 mg/mL CFS-oraCMS1 attenuated ERK phosphorylation (Figure 9B).

## 4. Discussion

Excessive osteoclast activity due to inflammation can lead to bone damage and loss, which can promote tissue damage and periodontal loss associated with periodontitis [3]. Therefore, if oral probiotics can inhibit the generation of osteoclasts, they are anticipated to suppress the occurrence and progression of periodontitis. Accordingly, several studies have evaluated probiotic therapeutic interventions as potential treatments to prevent alveolar bone loss.

These studies reported that probiotics inhibit bone loss in local inflammatory responses in conditions such as periodontitis, mostly using experimental animal models [16,17,18,26,27,28,31,32]. *Lactobacillus gasseri* and *Lactobacillus rhamnosus* were reported to reduce alveolar bone loss and inflammation scores in a periodontitis model induced by periodontal pathogens, including *P. gingivalis* [26,27]. In a ligation-induced rat periodontitis model, *Bifidobacterium animalis lactis*, *Bacillus subtilis*, and *Saccharomyces cerevisiae* were reported to reduce alveolar bone loss, reduce proinflammatory cytokines, lower osteoclast numbers, and alter the anaerobic–aerobic oral microbiota composition [31,32]. Additionally, *Lactobacillus brevis* and *W. cibaria* were reported to reduce alveolar bone loss, reduce proinflammatory cytokines, and reduce oral bacterial counts in a ligation-induced mouse periodontitis model [16,18].

Probiotics are defined as “live microorganisms that confer health benefits to the host when administered in adequate amounts” [33]. Although probiotics are being actively commercialized for their health benefits, technical limitations, such as viability control, place restrictions on production processes in various product development endeavors. Additionally, the safety profile of live strains when used in immunocompromised patients and pediatric populations has remained controversial [34,35].

Therefore, advancing research on functional foods in recent decades has given rise to a new conceptual term for microorganisms that have positive effects on their host. It has been reported that even non-viable microorganisms or the by-products of bacterial metabolism can exert biological activity in the host, leading to the emergence of new terms such as paraprobiotics and postbiotics [36]. Paraprobiotics are defined as “inactivated microbial cells that provide health benefits to the host” and include intact cell wall components with the cell structure intact. Postbiotics are defined as “probiotic metabolites that provide benefits to the host” and include CFSs containing proteins and organic acids secreted from viable cells [37].

CFSs from the postbiotics *Lactobacillus salivarius* and *Lactobacillus reuteri* have considerable potential as functional oral health ingredients to inhibit alveolar bone loss associated with periodontitis [25,38]. Meanwhile, the *W. cibaria* CMU (oraCMU) and CMS1 (oraCMS1) strains have been commercialized as oral probiotics, and there has been ongoing research on their beneficial effects on oral health [18,19,20,21,22]. In particular, oraCMU was confirmed to suppress alveolar bone loss in a periodontitis-induced animal model; however, research on other associated inhibitory mechanisms in addition to its anti-inflammatory mechanism has not yet been conducted [18]. Since there is a known correlation between osteoclasts and periodontitis, we sought to determine their inhibitory ability on osteoclastogenesis using HK bacteria as paraprobiotics as well as CFS-containing metabolites as postbiotics to gain a better understanding of the effect of oraCMU and oraCMS1 on osteoclastogenesis in vitro, in this study.

Osteoclasts are large, multinucleated cells that originate from hematopoietic stem cells [4,5,6]. Osteoclast differentiation is essentially regulated by RANKL [7,9]. RAW 264.7 cells are primarily used as osteoclast precursors because they differentiate into osteoclasts through RANKL stimulation [29,30]. RANK is present on the cell surface, and its expression is increased by RANKL, and differentiation into osteoclasts is promoted through the binding of RANK and RANKL. TRAP is abundantly distributed in the lysosomes of osteoclasts, and TRAP activity serves as a cytochemical marker that identifies multinucleated cells in bone tissue [39]. Therefore, in this study, TRAP staining was used to find out whether multinucleated osteoclasts were generated, and TRAP activity was measured as an indicator of the degree of osteoclast differentiation to investigate the effects of HK bacteria or metabolites.

We demonstrated that in the control group treated with RANKL alone, the formation of multinucleated osteoclasts was observed in RAW 264.7 cells, and treatment with both HK bacteria strains resulted in a significant dose-dependent reduction of both osteoclast formation and TRAP activity (Figure 2). Similarly, the CFSs also significantly reduced osteoclast formation and TRAP activity; however, even at a high concentration of 2 mg/mL, which did not exhibit cytotoxicity, its capacity to inhibit osteoclast formation was lower than that of HK bacteria (Figure 3). Therefore, it was confirmed that oraCMU and oraCMS1 suppress osteoclast differentiation by reducing TRAP activity, increased by RANKL, in both paraprobiotics and postbiotics.

Bone resorption activity is a typical characteristic of mature osteoclasts. Kyoi et al. [6] employed scanning electron microscopy to observe whether osteoclasts were formed on hard tissue sections to form microscopic pits on the surface. In our study, we evaluated pit formation using dentin discs to determine osteoclast function. The analysis revealed that the dentin disc surface of the untreated group was clean without pits, while the RANKL-only treated group exhibited larger pit sizes compared to the other treatment groups (Figure 4). These findings showed that osteoclasts can resorb bone and that the size of the bone pits increased proportionally with the number of osteoclasts. Analysis of the relative pit area revealed that it was significantly reduced in both the dead bacteria- and metabolite-treated groups. These findings suggest that both dead cells and metabolites effectively inhibit RANKL-mediated osteoclastogenesis and bone resorption.

Treating RAW 264.7 cells with RANKL initiates osteoclast differentiation signaling through RANK, thereby inducing the expression of key differentiation-related genes, including c-Fos, NFATc1, OSCAR, DC-STAMP, cathepsin K, and TRAP [7,8,9,10,11,12,13]. Furthermore, c-Fos and NFATc1 work synergistically to induce the expression of several key osteoclastogenesis-related genes [7,8,9,10]. Specifically, NFATc1 is known as a key transcriptional regulator of osteoclasts and is an essential factor for osteoclast differentiation [7,8,9,10]. OSCAR is an osteoclast-specific immune receptor that serves as a costimulatory signal essential for RANKL-mediated NFATc1 activation [11]. DC-STAMP is known to play a role in cell–cell fusion and is one of the fusion mediator molecules directly regulated by NFATc1 [13]. Meanwhile, cathepsin K and TRAP are cysteine proteases secreted by osteoclasts that are responsible for decomposing matrix proteins during bone resorption [12].

In this study, we found that HK-oraCMU and HK-oraCMS1 dose-dependently downregulated the gene expression levels of all analyzed osteoclast-specific genes, namely c-Fos, NFATc1, OSCAR, DC-STAMP, cathepsin K, and TRAP (Figure 5). Conversely, CFSs containing metabolites significantly suppressed gene expression levels for NFATc1, OSCAR, and cathepsin K but not TRAP, and this was observed for both strains (Figure 6). This is consistent with our findings that dead bacteria significantly downregulated all osteoclast-related genes, whereas metabolites only effectively downregulated certain genes. This result supports the fact that, when comparing TRAP results, dead bacteria exhibited a better inhibitory effect on osteoclast differentiation than metabolites. These results suggest that dead cells and their metabolites regulate osteoclastogenesis at different transcriptional levels.

Additionally, this study confirmed that dead bacteria significantly reduced the expression of all proteins dose-dependently, which is consistent with the results of gene expression inhibition (Figure 7). To determine the effect of direct interaction between HK bacteria and host cells, cell culture inserts with a pore size of 0.4 μm were used to prevent contact between RAW 264.7 cells and dead cells. Notably, HK bacteria did not inhibit the expression of proteins associated with osteoclast differentiation (Figure 7). These experiments confirmed that HK-oraCMU and HK-oraCMS1 require direct interaction with osteoclast precursors to inhibit osteoclastogenesis. These results are consistent with those of a previous study in which live *W. cibaria* CMU showed an anti-inflammatory effect on human gingival fibroblasts through direct contact [22]. Similarly, metabolites demonstrated a tendency to reduce the expression of proteins associated with osteoclast differentiation, with high concentrations of CFS-oraCMS1 inducing a significant decrease, which is consistent with the findings on gene expression analysis (Figure 8). Therefore, our results suggest that oraCMU and oraCMS1 inhibit osteoclastogenesis by downregulating the expression of proteins involved in osteoclast bone resorption and suppressing the expression of key downstream osteoclast differentiation-related genes, including NFATc1.

Osteoclast differentiation, mediated by RANKL/RANK binding, is achieved through a specific intracellular signaling pathway. Specifically, intracellular NF-κB and MAPK are activated, leading to increased expression of the transcription factor c-Fos, and subsequently, NFATc1, a crucial transcription factor for the entire osteoclast differentiation and formation process, is activated, resulting in osteoclast differentiation [4,9,10]. In this study, we confirmed through Western blotting analysis that HK bacteria and CFSs downregulate the phosphorylation of JNK and p38 during RANKL-induced osteoclast differentiation. These results suggest that oraCMU and oraCMS1 inhibit RANKL-induced osteoclastogenesis by blocking the MAPK signaling pathways, thereby downregulating the expression of the essential transcription factors c-Fos and NFATc1, subsequently downregulating various downstream-related genes including OSCAR, DC-STAMP, cathepsin K, and TRAP.

Taken together, our results demonstrated that paraprobiotic-like dead cells suppress osteoclastogenesis more effectively than postbiotic-like metabolites. Additionally, this study revealed that the dead bacterial strains CMU and CMS1 inhibited osteoclastogenesis through direct interaction with the host cells, which inactivated the MAPK signaling pathways, including JNK and p38, subsequently downregulating osteoclast-related genes. This suggests that periodontitis can be prevented by suppressing the expression of osteoclast-associated proteins, resulting in the suppression of osteoclast differentiation and bone resorption.

Paraprobiotics primarily reside in the bacterial cell envelope, encompassing a variety of molecules, including peptidoglycan, teichoic acid, cell wall polysaccharides, and cell surface-related proteins. These components are crucial effector molecules since they are the initial points of interaction with host cells [37]. Conversely, postbiotics refer to substances that bacteria secrete or release into the host environment following bacterial lysis, offering various physiological benefits to the host, including proteins, organic acids, and peptides [37]. However, this study has a few limitations. First, it was not possible to secure a sample size that could statistically determine the mechanism by which the HK bacteria and CFSs inactivate the NF-κB and MAPK pathways. Second, this study could not elucidate why CFS-oraCMS1 shows differentiation from other tested substances by downregulating ERK phosphorylation. Therefore, further research is needed in this study to specifically discuss strain-specific mechanisms of osteoclast formation inhibition in HK bacteria and CFSs.

## 5. Conclusions

The *W. cibaria* strains CMU and CMS1 can prevent periodontitis by suppressing osteoclast differentiation by inhibiting the expression of osteoclast-associated genes and proteins, thereby preventing bone destruction. Further research is needed to elucidate the effective in vitro components and modes of action of these bacteria against osteoclastogenesis.

## Data Availability

The data presented in this study are available upon request from the corresponding author.
